# Editorial: Advanced sensing, learning and control for effective human-robot interaction

**DOI:** 10.3389/frobt.2026.1928993

**Published:** 2026-07-24

**Authors:** Zhenyu Lu, Lu Chen, Yanpeng Guan, Jing Luo, Chao Zeng

**Affiliations:** 1 Bristol Robotics Laboratory, Bristol, United Kingdom; 2 South China University of Technology, Guangzhou, China; 3 Shanxi University, Taiyuan, China; 4 Wuhan University of Technology, Wuhan, China

**Keywords:** control, human robot interaction, learning, sensing, summary

Robotics is increasingly moving toward a paradigm in which machines are no longer viewed merely as tools, but as intelligent partners capable of perceiving and acting within dynamic, unstructured human environments. Achieving this transition, however, requires more than improved actuation or task execution. Robots must acquire reliable information about people and environments, transform sensor data into task-relevant perceptual representations, learn from feedback and experience, and convert these representations into safe and efficient control actions. Accordingly, advances in sensor design, high-level perception, learning, and control have improved robots’ ability to infer human intent. They also allow robotic systems to adapt to user preferences, environmental changes, and incomplete prior knowledge while balancing safety constraints with operational efficiency. Methods such as adaptive querying further strengthen human feedback loops, enabling robots to align their behavior with user expectations and collaborate more effectively. Centered on the theme of “*Advanced Sensing, Learning and Control for Effective Human-Robot Interaction,*” this Research Topic focuses on the system-level integration of these capabilities rather than on isolated algorithmic advances. It highlights how sensing, learning, and control can be combined to close the loop between human input, environmental perception, robot decision-making, and physical execution. The papers collected here examine this Research Topic across human-in-the-loop robot learning, robust long-horizon action recognition, intelligent agricultural perception, and autonomous medical scanning. Together, they contribute to the development of robotic systems with improved situational awareness, responsiveness, and safety in human-centered environments.

Against this background, this Special Issue brings together four contributions that illustrate recent progress in human-robot interaction through the integration of sensing, learning, and control. In “Adaptive querying for reward learning from human feedback,” Anand et al. examine how robots can actively select informative query states and appropriate feedback formats when learning from users. Their two-stage framework treats feedback acquisition as an optimization problem, jointly considering state informativeness and feedback format rather than relying on a fixed supervision signal. This approach improves sample efficiency and supports the learning of human-aligned and safe behavior. In a complementary direction, “Bayesian Selective Scan for Robust Long-Horizon Action Recognition via Reliability-Aware Discretization” by Wang focuses on robustness in temporal perception. The proposed reliability-aware selective state-space model incorporates Bayesian uncertainty into temporal modeling, improving tolerance to corrupted or unreliable inputs while preserving long-horizon memory. This capability is important for continuous interaction, where unreliable visual segments can otherwise affect long-term action representations and downstream recognition or decision-making.

The remaining contributions shift the discussion from interaction-level learning and temporal reliability to efficient perception and closed-loop control in applied robotic systems. In “*Enhancing weed detection through knowledge distillation and attention mechanism,*” El Alaoui and Mousannif address precision agricultural robotics, where visual perception must remain reliable under constraints on model size, latency, and computational resources. Their knowledge distillation framework transfers the representational capacity of deep visual models to a compact architecture, while attention mechanisms help preserve features relevant to weed detection. In this setting, model compression becomes a practical requirement for deploying advanced perception algorithms in field robotics. The medical robotics contribution shifts the focus to a safety-critical clinical environment, where sensing, planning, and control must support real-time adjustment. In “*Autonomous ultrasound scanning robotic system based on human posture recognition and image servo control: an application for cardiac imaging,*” Tang et al. present a cardiac imaging system that integrates human posture recognition, point-cloud perception, autonomous scan-path planning, image servo control, and adaptive admittance control for probe adjustment and image-quality optimization. The resulting closed-loop scanning pipeline shows how advanced sensing and control can reduce operator dependence, improve procedural reproducibility, and support more reliable human-centered assistance in medical imaging.

Taken together, these papers reflect a maturing field in which multidisciplinary methods are helping to bridge theoretical research and practical deployment in real-world human environments. Although their application domains differ, these studies converge on a common problem: how robots can acquire reliable information in uncertain environments, interpret task requirements and human preferences, make adaptive decisions, and complete tasks through real-time and stable control. They therefore suggest that effective human-robot interaction does not depend on a single algorithmic component. Instead, it emerges from the coordinated integration of sensing, learning, decision-making, and control. Continued research in this direction will remain essential for translating human-centered robotic systems from controlled experimental settings into dependable applications in everyday, agricultural, and clinical environments.

